# Assessing cognitive flexibility in mice using a custom-built touchscreen chamber

**DOI:** 10.3389/fnbeh.2025.1536458

**Published:** 2025-02-13

**Authors:** Rui C. Pais, Ali Goldani, Jayden Hutchison, Amirhossein Mazrouei, Mostafa Khavaninzadeh, Leonardo A. Molina, Robert J. Sutherland, Majid H. Mohajerani

**Affiliations:** ^1^Department of Neuroscience, Canadian Centre for Behavioural Neuroscience, University of Lethbridge, Lethbridge, AB, Canada; ^2^Cumming School of Medicine Optogenetics Core Facility, Cumming School of Medicine, University of Calgary, Calgary, AB, Canada; ^3^Department of Psychiatry, Douglas Hospital Research Centre, McGill University, Montréal, QC, Canada

**Keywords:** touchscreen chamber, Raspberry Pi, serial reversal learning, cognitive flexibility, Alzheimer’s disease

## Abstract

Automated touchscreen systems have become increasingly prevalent in rodent model screening. This technology has significantly enhanced cognitive and behavioral assessments in mice and has bridged the translational gap between basic research using rodent models and human clinical research. Our study introduces a custom-built touchscreen operant conditioning chamber powered by a Raspberry Pi and a commercially available computer tablet, which effectively addresses the significant cost barriers traditionally associated with this technology. In order to test our prototype, we decided to train C57BL/6 mice on a visual discrimination serial-reversal task, and both C57BL/6 and App^NL−G−F^strain - an Alzheimer’s Disease (AD) mouse model - on a new location discrimination serial-reversal task. The results demonstrated a clear progression toward asymptotic performance, particularly in the location discrimination task, which also revealed potential genotype-specific deficits, with App^NL−G−F^ mice displaying an increase in the average number of errors in the first reversal as well as in perseverative errors, compared to wild-type mice. These results validate the practical utility of our touchscreen apparatus and underline its potential to provide insights into the behavioral and cognitive markers of neurobiological disorders.

## 1 Introduction

The evolution of behavioral tasks in Neuroscience, from traditional mazes to touchscreen paradigms, has yielded profound insights about the dynamic interplay between brain and behavior. The development and refinement of rodent touchscreen chambers, as well as the wide variety of tasks developed for this platform over the years, has been remarkably successful in evaluating different cognitive skills in both wild-type and genetically modified rodent strains, as well as in the ability to investigate potential behavioral and neurophysiological changes resulting from pharmacological interventions (Bussey et al., 1994; Bussey et al., 1997; Bussey et al., 2001; Talpos et al., 2009; Horner et al., 2013; Mar et al., 2013; Hvoslef-Eide et al., 2016; Nilsson et al., 2016).

Ever since Skinner’s groundbreaking work in the context of reflexive physiology introduced automated training in the 1930’s, researchers have uncovered a plethora of tools for understanding learning processes (Skinner, 1937, 1986; Staddon and Cerutti, 2003). The development of operant conditioning boxes allowed for the precise manipulation of contextual contingencies and the measurement of behavior over a specified period of time, and significantly reduced the interaction between the experimenters and the animal subjects (Ferster, 1953; Weiss, 1972; Wetzel, 1986; Staddon and Cerutti, 2003; Mar et al., 2013; Sakagami and Lattal, 2016; Pinkston, 2022). By using levers or buttons the animals can press or peck in order to obtain a reinforcement (e.g., water, food pellets among others), the involvement of the experimenter during training is minimized, in favor of an auto-shaping process whereby the animals can learn the desired behaviors independently.

These operant conditioning apparatuses continued to evolve, and over time researchers started to incorporate computer screens where different images were displayed, and eventually touchscreen systems, which allowed the animals to directly interact with the displayed images in order to make a choice. The touchscreen chambers, which were initially developed to be used with pigeons, as well as human and non-human primates, were eventually adapted for rodents in the mid-nineties, and have become an invaluable tool in cognitive and behavioral neuroscience research since then (Wright et al., 1988; Bussey et al., 1994; Markham et al., 1996; Bussey et al., 2001; Izquierdo et al., 2006; Bussey et al., 2008; Winters et al., 2008; Mar et al., 2013; Nithianantharajah et al., 2015; Nilsson et al., 2016; Sakagami and Lattal, 2016; Phillips et al., 2017; Sullivan, 2022). In comparison to more traditional approaches to rodent phenotyping methods, which require multiple tests in different environments such as open-fields, mazes or conventional operant conditioning boxes, the touchscreen technology offers a controlled setting that closely mimics human cognitive assessment. This allows not only for more accurate data collection, but also for a significantly less stressful experience for the animals (O’Leary et al., 2018; Dumont et al., 2021; Sullivan, 2022).

Over the years, researchers have developed multiple tasks that cover a wide range of cognitive functions, such as visual discrimination, object-location paired-associations, visual-category learning, working memory, rule-switching, or pattern separation tasks (Hvoslef-Eide et al., 2015, 2016; Kim et al., 2015, 2016; Kwak et al., 2015, 2016; Creighton et al., 2019; Barnard et al., 2021; Wang et al., 2022). In addition, the touchscreen chamber enables high throughput testing by allowing multiple animals to be tested simultaneously; effectively streamlining the efficiency of the experimental procedures and allowing experiments to be conducted as required. With its high degree of automation, similarities to human-based cognitive assessments, and the standardization of touchscreen tasks, this behavioral apparatus has enhanced the translatability of preclinical models, leading to its widespread adoption across multiple research institutions. These include universities, biotechnological firms, and pharmaceutical companies, particularly as mice have become the preferred model organism in basic and preclinical research, due to the widespread availability of transgenic lines and the continuous refinement of genetic and molecular tools that enable *in vivo* recordings and circuit labeling (Dickson et al., 2013; Horner et al., 2013; Hvoslef-Eide et al., 2016; Dumont et al., 2021).

Among the different applications of this technology, reversal learning tasks have emerged as an important tool for assessing cognitive flexibility. These tasks require multiple executive functions such as attention, working memory or response inhibition, and depend on the subjects’ adaptability to changing rewards or feedback (Fowler, 1980; Cools et al., 2002; Dickson et al., 2013; Bryce and Howland, 2015; Izquierdo et al., 2017; Marquardt et al., 2017; Van den Broeck et al., 2019; Odland et al., 2021). Serial reversal paradigms further test the ability to learn, maintain, and then re-learn behavioral rules over multiple iterations, as each change requires the suppression of previously reinforced behaviors and the subsequent adaptation to new rules, thus engaging executive functions such as inhibitory control, cognitive flexibility and attentional processes to an even greater extent (Boulougouris et al., 2007; Castañé Anna et al., 2010; Kosaki and Watanabe, 2012; Dickson et al., 2013; Izquierdo et al., 2017).

Reversal learning studies were among the first to adopt touchscreen technology for both human and non-human primates, whereas rodent studies typically relied on either spatial or non-visual cues - a discrepancy that stemmed from automation challenges and difficulties in standardizing experiments across species. However, touchscreen technology has bridged this gap and enabled standardized tasks that could be adapted and used across various species, while maintaining the underlying focus on adaptive responses and rule switching (Bussey et al., 1997; Bussey et al., 2001; Talpos et al., 2009; Hvoslef-Eide et al., 2015, 2016; Nithianantharajah et al., 2015; Nilsson et al., 2016).

Despite its longstanding use, reversal learning remains an important behavioral paradigm, especially when it comes to identifying learning and cognitive flexibility deficits in neuropsychiatric disorders, such as schizophrenia, obsessive-compulsive disorder (OCD), depression, autism, Parkinson’s, and Alzheimer’s disease (Lafleche and Albert, 1995; Valerius et al., 2008; Wobrock et al., 2009; Marazziti et al., 2010; Millan et al., 2012; D’Cruz et al., 2013; Gruner and Pittenger, 2017; Guarino et al., 2019; Jara-Rizzo et al., 2020; Monni et al., 2023). Concurrently, cross-species studies have also highlighted the role of the prefrontal cortex - specifically, the orbitofrontal (OFC) and medial prefrontal (mPFC) cortices – as well as subcortical regions such as the dorsal striatum and amygdala, in facilitating these tasks (Dias et al., 1996; Cools et al., 2002; Chudasama and Robbins, 2003; Hornak et al., 2004; Hampshire and Owen, 2006; Izquierdo et al., 2006, 2017; Clatworthy et al., 2009; Brigman et al., 2010; Graybeal et al., 2011; Izquierdo and Jentsch, 2012; Lucantonio et al., 2014; Alsiö et al., 2015).

While the benefits of touchscreen-based tasks for assessing cognitive and behavioral skills in rodents, and more specifically mice, are clear, especially in bridging the gap between species through standardized procedures, the adoption of these technologies is not without its challenges. Despite its numerous advantages, the main concern regarding the adoption of rodent touchscreen chambers has remained relatively unchanged over the years, and that is the considerable financial investment required. The expenses associated with acquiring even a single exemplar of these touchscreen chambers can be prohibitively high, which effectively hinders an even more widespread adoption and a swifter integration into the arsenal of behavioral assessment tools in basic research. Even though this technology has become progressively less expensive, the large financial outlay has led different research groups to develop their own alternatives to circumvent this issue (Pineño, 2014; O’Leary et al., 2018; Wiesbrock et al., 2022; Eleftheriou et al., 2023). This is particularly notable considering the accessibility of modern touchscreens as well as the different components required for the assembly and functioning of a similar product, which allow for the development and programming of various touchscreen-based tasks tailored to specific research needs.

Driven by the evolving demands of cognitive and behavioral neuroscience for automated and adaptable experimental tools, alongside the practical challenges of high equipment costs, and the need to collect behaviorally relevant data on both wild-type and Alzheimer’s disease mouse models, we set out to develop a custom touchscreen apparatus for mice. To validate this approach, we designed and implemented two distinct touchscreen tasks with a specific focus on cognitive flexibility: a visual discrimination serial-reversal task, and a location discrimination serial-reversal task.

Our efforts reflect a need to develop versatile and accessible technologies to advance research in rodent cognitive flexibility, and ultimately contribute to a broader comprehension of both normal and pathological brain functions.

## 2 Materials and methods

### 2.1 Hardware

**FIGURE 1:**
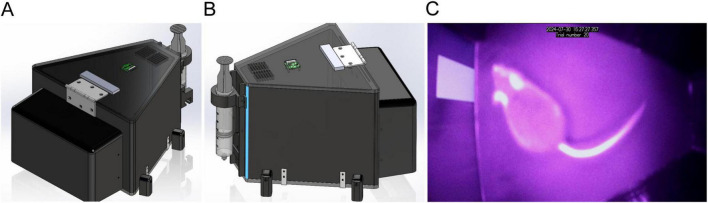
Custom-built touchscreen chamber for mouse behavioral studies. The tree-quarter **(A)** and side profile **(B)** views of the touchscreen chamber, highlighting the integrated design features and structural components. **(C)** Interior view of the chamber during a pre-training session of the location discrimination serial-reversal task, with a blinking cue on the right side of the screen.

The touchscreen apparatus was designed using computer-aided design software (SOLIDWORKS 2023 SP 3.0, Dassault Systèmes) and was adapted from specifications detailed in prior studies (Horner et al., 2013), as seen in Figures 1A, B. The inner chamber featured a trapezoidal behavioral area, or more accurately, a triangle with rounded corners, optimized to focus on both the touchscreen and the reward area. Specific dimensions were 80 mm wide at the reward area, 260 mm wide at the screen, and a trapezoidal length of 240 mm, with a working area height of 190 mm and wall thicknesses of 10 mm. The walls were 3D printed using black PLA to minimize external light interference and enhance visual contrast during experiments. The lid and floor of the chamber were constructed from 6.5 mm thick black plexiglass to facilitate cleaning and maintain durability.

For the touchscreen interface we selected a Samsung Galaxy Tab A 8.0 SM-T350 (Samsung Electronics Co., Ltd.), with a resolution of 1,024 × 768 pixels, mounted horizontally opposite the reward area and accessed through a 163 × 125 mm aperture. This tablet not only recorded touch interactions but also managed the experimental flow, communicating with a Raspberry Pi (RPi) 4 Model B (8 GB RAM). The Raspberry Pi was enclosed in a custom 3D-printed case attached to the touchscreen wall, designed with apertures for cable management and component interconnection.

Reward delivery was managed using a 5V solenoid valve connected to medical-grade silicone tubing (HelixMark Standard Silicone Tubing, Freudenberg Medical), which extended to a metal tube. This tube, protruding 10 mm from the wall, was 3 mm in diameter and dispensed approximately 2.5 μl of 10% sucrose water. The sucrose solution was stored in a 60 ml syringe, functioning as the reservoir for the system. The availability of the reward was signaled by a blue LED visible through a 3 mm round aperture, positioned 10 mm above the reward tube, and auditory cues that varied by the type of response were emitted through the tablet’s speakers.

Videos were recorded by a small camera (Raspberry Pi Camera Module 2), positioned on top of the lid, to capture detailed activity within the chamber, and enhanced by an array of infrared LEDs for consistent illumination under low lighting conditions (Figure 1C). This setup not only allowed the videos to be recorded locally on the RPi for later analysis, but also enabled the hosting of a local live stream from inside the chamber as soon as the trial software started. This annotated live stream allowed experimenters to supervise real-time activity within the chamber and address any issues that might interfere with the flow of the experiment.

### 2.2 Software

**FIGURE 2:**
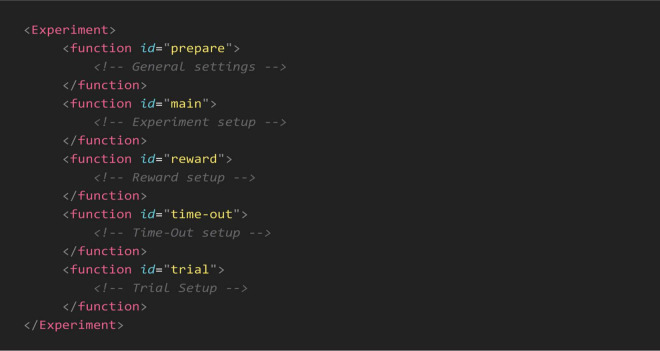
General outline of an XML configuration file. Each configuration file is divided into sections, enclosed within specific “function” tags, to control task parameters. The “prepare” function defines general settings, including session duration, screen sections, initial rewards, and pre-loaded images or virtual objects. The “main” function sets the trial loop by specifying the number of trials per session. Task outcomes are adjusted by the “reward” and “time-out” functions, which configure audio-visual feedback and reward delivery based on the animal’s responses. The “trial” function specifies trial parameters, such as visual cues, object dimensions and valence for each learning stage. All parameters are fully customizable to meet experimental requirements.

To give researchers the ability to create and control task parameters, we used an XML schema to define each experiment’s specifications. An XML configuration file for an experiment is structured with tags that define different functions and sections of the experiment. Each function or parameter is enclosed in and may have various attributes. The general outline of a configuration file is shown in Figure 2.

There are five main functions within each configuration file for setting up the experimental environment. The *prepare* function allows experimenters to specify key parameters: (1) overall duration, which dictates that the experiment continues until either completion or the specified duration elapses; (2) background color, which defines the visual setting of the experiment; (3) number and size of sections, determining whether the active touching area is divided into two or four sections; (4) section dividers, specifying both the presence and color of dividers between sections; (5) initial reward cues, including the presence, number, and timing interval between these cues; (6) touch time-out, setting the duration before a time-out is triggered when the wrong image/3D object or side of the screen is touched; (7) image pre-loading, which minimizes the image/3D object load times during the experiment.

Within the *main* function, experimenters can specify the number of trials, setting it to a predetermined amount based on their experimental design. In the *reward* function, users can specify a text for logging in the final reports whenever the reward is triggered, adjust the frequency and duration of the tone played, and control the opening and closing durations of the solenoid valve. Similar to the *reward* function, the *time-out* function allows for the display of a time-out alert by filling the entire screen with a bright color for a specified duration. Users can also determine the sections where the correct and incorrect images appear; if not specified, experimenters can choose to randomize the location for each trial.

Finally, in the *trial* function, experimenters can define each trial’s parameters. For visual discrimination tasks, they can select a single image or 3D virtual object or allow a random choice from a series of images for both rewarded (S+) and unrewarded (S-) categories. In location discrimination tasks, the settings allow for a cue to be set to static or blinking, with adjustable frequency. This configuration syntax enables experimenters to create a diverse range of touchscreen tasks tailored to their research needs.

**FIGURE 3:**
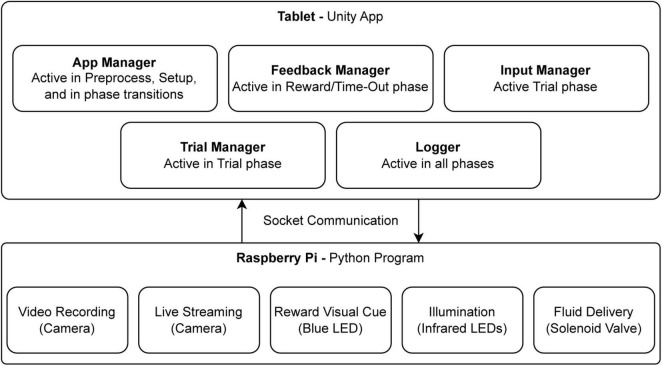
The main software and hardware components. The diagram illustrates the interaction between software components, running on a tablet via Unity, and hardware components controlled by a Python program on a Raspberry Pi. The Unity application comprises several modules, each operating in distinct event loops during specific trials phases: the App Manager (preprocessing, setup, and transitions), Feedback Manager (reward and time-out phases), Input Manager (trial phase), Trial Manager (trial control), and Logger (data recording across all phases). Communication between software modules and hardware is achieved through socket communication. The Raspberry Pi program manages video recording, live streaming, visual cues (blue LED for rewards), infrared illumination, and fluid delivery via a solenoid valve.

The software deployed on the Samsung tablet is a Unity application developed with Unity Game Engine (Unity Technologies, 2024). Through Unity, we could easily develop the logic of the software and, using its tools for building Graphical User Interface (GUI), create the interface that best suits the experimenter’s needs. To communicate with the RPi and to be able to control the hardware modules, we implemented a socket communication system so the tablet can send commands to the RPi through a wireless network. RPi’s built-in GPIO4 and Picamera5 libraries were used for communication with the hardware. The software is developed as a state machine with main components working in their own evet loops. An overall view of the software components is shown in Figure 3.

The software running on the RPi is a python program that hosts a socket server and accepts connections from the tablet running the Unity app. Through this socket communication, commands from the tablet are sent with minimum delay to control hardware components connected to the RPi. For example, when the socket server receives the command “reward,” it turns on the blue LED and opens the Solenoid Valve for a split second to deliver reward fluid.

**FIGURE 4:**
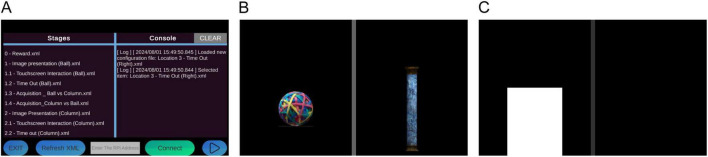
Screenshots of the software. **(A)** Main menu of the program, where the user gets to choose the configuration file (learning stage) for the experiment. Configuring a connection to the RPi controller is also established in this page; the user inputs the network address of the RPi and initiates the connection. **(B)** Screenshot of a 2-section visual discrimination task. **(C)** Screenshot of a 2-section location task during pre-training, where a blinking cue appears on the screen to signal the S+ location.

Screenshots from the Unity app can be found in Figure 4. The source code for the software part of this project can be found on our GitHub page.

### 2.3 Experimental flow

The experiments performed with this software follow a general pattern. Each experiment starts with initial reward deliveries to give some satiation to the animals before the actual trials start. One can select multiple or no initial rewards. Then the program proceeds to execute the trials as defined by the user; they can be any kind of trial explainable by the options provided in XML configuration files.

To enhance engagement and ensure variety, object and cue placements during each trial are randomized using the System Random library in C#. This method pseudo-randomly shuffles indices representing positions, ensuring objects appear in different locations across trials. The random generator avoids using a fixed seed value to prevent the sessions from becoming repetitive. Additionally, a safeguard is implemented to limit repetitive placement patterns to no more than three consecutive trials. This measure minimizes the emergence of patterns that could inadvertently bias behavior while maintaining a balance between randomness and controlled variety across learning stages.

**FIGURE 5:**
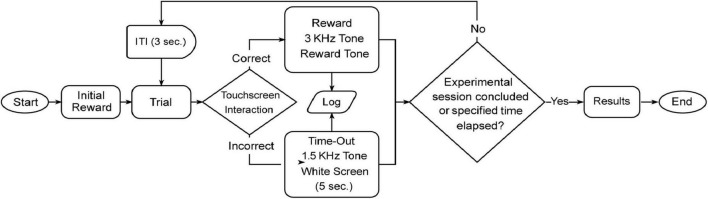
Overview of a typical experimental session. The flowchart depicts the sequence of events during an experimental session, starting from the initial reward to mark the beginning of the task for the animals. After an inter-trial interval (ITI) of 3 s, the mouse interacts with the touchscreen during the trial phase. Trial outcomes are determined by response accuracy: correct responses result in a reward paired with a 3 kHz tone, while incorrect responses trigger a time-out, signaled by a 1.5 kHz tone and a white screen displayed for 5 s. Each trial is logged, and the session continues until the predefined duration is reached or the experiment concludes. Upon completion, results are saved and displayed on the touchscreen, marking the end of the session.

All the activities of the subject are recorded from this point, any interaction with trial objects that results in a feedback response, will be logged in a .CSV report file, accessible at the end of the experiment. Furthermore, the video recording will capture all the ongoing events within the experiment box and contains timestamps of the screen interactions along with their respective outcomes (time-out or rewarded), as well as trial number. The flow of the experiment can be seen more clearly in Figure 5.

### 2.4 Subjects

A total of 27 adult mice, bred in-house, were used in this study: 9 C57BL/6 mice (23–31 g, 6–8 months old, three males and six females) for the object reversal learning task, and 18 mice, comprising 9 C57BL/6 mice (26–31 g, 8–9 months old, four males and five females) and 9 App^NL−G−F^ knock-in mice (25–32 g, 8–10 months old, four males and five females) for the location-reversal task.

All animals were housed in groups of 2–4 individuals, in standard mouse cages. The room temperature was maintained at 24°C under a 12 h light/dark cycle with the lights on at 7:30 AM and free access to food and water before the beginning of the behavioral training. All procedures were in accordance with the guidelines established by the Canadian Council on Animal care and with the protocols approved by the Animal Welfare Committee of the University of Lethbridge.

Mice were water deprived throughout the duration of the behavioral training. During this period mice were given a daily *ad libitum* access to water for 30 min in their home cages 30 min after the last training session, and their weight was maintained to at least 85% of the baseline. All mice were carefully monitored daily to ensure their well-being. During the water restriction period, which spanned the duration of the experiment, mice were weighed twice daily, and no signs of distress were observed in any of the mice.

#### 2.4.1 Alzheimer’s disease mouse model

Alzheimer’s Disease (AD) is the most prevalent form of dementia, and it is characterized by the progressive aggregation of amyloid-β (Aβ) and formation of neurofibrillary tangles, which lead to memory loss, cognitive impairments, and overall decline in quality of life (Braak and Braak, 1991; Ettcheto et al., 2018; Folch et al., 2018; Mehla et al., 2019; McAllister et al., 2020). Central to AD pathogenesis is the spread of Aβ, resulting in neuroinflammation, plaque deposition, and tau hyperphosphorylation, which eventually causes brain atrophy (Harper and Lansbury, 1997; Bloom, 2014; Walker et al., 2018).

The App^NL−G−F^ mouse model used in this study, incorporates humanized murine Aβ sequences with three specific mutations: Swedish (NL), Beyreuther/Iberian (F), and Arctic (G) (Nilsson et al., 2014; Saito et al., 2014). Unlike other App transgenic lines, the App^NL−G−F^ model avoids artifacts introduced by App overexpression by using a knock-in approach to express App at wild-type levels, thus ensuring that any observed pathologies are a direct result of pathogenic Aβ rather than App overexpression (Guardia-Laguarta et al., 2010; Shin et al., 2010; Saito et al., 2014). This mouse model expresses App with familial Alzheimer’s disease-associated mutations which promote Aβ toxicity, an increase in total Aβ production, the Aβ42/Aβ40 ratio, as well as promoting Aβ aggregation (Saito et al., 2014). In addition, this model reproduces several pathologies associated with AD including amyloid plaques, synaptic loss, and neuroinflammation - specifically microgliosis and astrocytosis around plaques - while also displaying age-associated cognitive impairments that can be observed as early as 6 months of age in some behavioral paradigms (Saito et al., 2014; Latif-Hernandez et al., 2019, 2020; Upîte et al., 2020; Lacoursiere et al., 2022; Mehla et al., 2023).

### 2.5 Experimental design

#### 2.5.1 Visual discriminating serial-reversal task

This task is based on the classic touchscreen pairwise discrimination task described in previous studies (Horner et al., 2013; Mar et al., 2013), with some slight modifications. Briefly, in this task mice need to choose between two images, or virtual objects, appearing on each side of the screen, by touching the surface of the touchscreen where the virtual objects are displayed. Before the pairwise discrimination takes place, the animals need to undergo some form of pretraining, where they learn the basic rules of the task in a progressive stepwise manner. The pre-training sessions were divided into four different stages: (1) *Habituation*, in which mice are introduced to the touchscreen chamber for 10 and 30 min, for two consecutive days, followed by two daily sessions of 60 min each, where the screen is OFF and the reward is delivered in 10 s intervals; (2) *Image Presentation*, where the rewarded (S+) image is introduced and paired with a tone and the reward delivery in 10 s intervals, for a total of 60 min; (3) *Touchscreen Interaction*, where the animals must learn to touch the area on the screen where the object appears in order to trigger the release of the reward for a total of 30 trials or 60 min duration; (4) *Time-Out*, where mice are introduced to a small time-out on commission of an error, if the screen is touched anywhere besides where the S+ image appears, with the passing criteria defined as 80% correct responses or 24 out of 30 trials for two consecutive sessions. Finally, in the *Acquisition* stage, the S- image is introduced, and mice must make a choice between the S+ and S- images which can appear on either the left or right side of the screen in a pseudo-random manner. After completing this stage, the reward contingencies are then reversed, and the S+ becomes the new S- and vice-versa. This cycle is then repeated five times, with an upper limit of 60 sessions per reversal.

To minimize the total time required to complete the task, mice were typically trained 2–3 times per day (once in the morning and 1–2 times in the afternoon). If a mouse demonstrated slower progress or lower motivation during the second session of the day, we limited training to 2 sessions to avoid overburdening the animals. Most mice, however, performed well with 3 sessions per day, which ensured consistent task exposure while maintaining welfare standards.

#### 2.5.2 Location discrimination serial-reversal task

The location discrimination reversal task we developed differs from the one used in previous studies (Kim et al., 2015; Saifullah et al., 2020), in the sense that it essentially functions as the mirror image of the visual discrimination task. Instead of using a two-phase task with low and high degrees of separation between stimuli comprised of bright squares, we decided to take advantage of the animals’ tendency to persevere after a correct choice. In other words, instead of having several within-session location-reversals, we opted for having a reversal-learning scheme across sessions, where we allowed mice to essentially become “sided” and then once the passing criteria is reached (> 80% correct responses), we reverse the contingency, making the previously unrewarded side of the screen (S-), the new S+. In this task we also used the same images used in the visual discrimination task, but now they serve as distractors which mice need to ignore and focus only on the side of the screen that correspond to the S+. The pretraining sessions followed a similar structure to the the visual discrimination task, with a few notable differences.

The task starts with the (1) *Habituation* stage, which follows the same parameters described in the visual discrimination task. In the (2) *Cue Presentation* stage, a blinking cue (1x per second) appears on either the left or right side of the screen (depending on the starting location determined *a priori* by the experimenter) signaling the S+ location. The following pre-training stages – *Touchscreen Interaction* (3) and *Time-Out* (4) – follow the exact same criteria outlined in the previous task. In the 4*^th^* and the last stage of pre-training (*Pre-acquisition*), the blinking cue is eliminated, and we introduce two distractor images, the same ones used in the visual discrimination task, but here, only one of them can appear in a pseudo-random fashion, on each trial. The animals must ignore the distractor image and continue to touch the same side of the screen to obtain the reward. Finally, in the *Acquisition* stage, both distractor images are presented on either side of the screen in a pseudo-random manner across trials. The objective is for the animals to consistently select the S+ side of the screen. The contingencies are then reversed five times, with the S+ and S- switching between the right and left side of the screen at each reversal, with the passing criteria remaining at 80% correct responses.

Mice were trained twice daily, once in the morning and once in the afternoon, on a consistent schedule that supported task acquisition while sustaining their motivation and overall condition.

### 2.6 Data analysis

Behavioral performance was monitored through post-session video analysis. The data from each session were automatically saved as .CSV files, organized in Microsoft Excel (Office 2021), analyzed using GraphPad Prism (GraphPad Software Inc. Version 10.2.3), and the figures prepared using Adobe Illustrator (Adobe Systems Inc. Version 27.8.1).

Statistical analyses were conducted using ANOVAs, with a significance threshold set at *p* < 0.05. Paired *t*-tests were used to compare error types within each reversal of the visual discrimination serial-reversal task. For the analysis of error types in the first reversal of the location discrimination task, a mixed-effects model (REML) was employed, with Fisher’s LSD test used for *post hoc* comparisons.

## 3 Results

### 3.1 Visual discrimination task

The visual discrimination serial-reversal task proved to be a demanding cognitive challenge for C57 mice, with completion time showing notable variability across subjects. On average, mice required M = 64.77 days (SD = 13.30) to achieve task proficiency, with completion times ranging from 48 to 89 days.

#### 3.1.1 Average number of sessions

**FIGURE 6:**
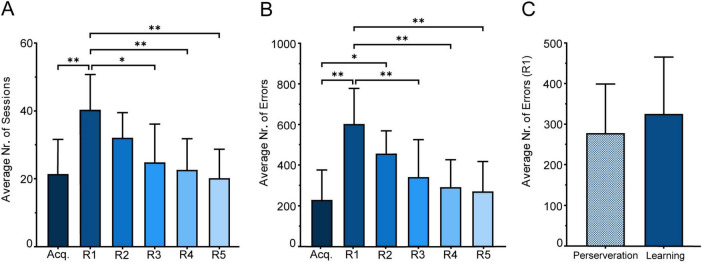
Performance in the visual discrimination serial-reversal task. **(A)** Average number of sessions across all learning stages. **(B)** Average number of errors across all learning stages. **(C)** Comparison between perseverance errors (sessions with ≤ 45% correct responses) and learning errors (errors in sessions with performance above 45%) in R1. Mean (M) ± SD in each learning stage. Statistical significance indicated as *p < 0.05, **p < 0.001.

The number of sessions required to complete the experiment varied across learning stages (Figure 6A), with means and standard deviations as follows: Acquisition (Acq.) phase had M = 21.44 (SD = 10.13), while Reversal 1 (R1) increased to M = 40.44 (SD = 10.30), with subsequent learning stages (R2 through R5) showing a gradual decrease in session counts. Specifically, R2 had an M = 32.11 (SD = 7.39), R3 an M = 24.89 (SD = 11.24), R4 an M = 22.67 (SD = 9.08), and R5 an M = 20.22 (SD = 8.45).

In order to assess performance differences across stages, a Repeated Measures One-Way ANOVA with Geisser-Greenhouse correction (ε = 0.6690), revealed significant variability among the session means, F(3.345, 26.76) = 7.942, *p* < 0.001. Tukey’s multiple comparison test further identified significant differences between the Acquisition phase (Acq) and the first Reversal stage (R1), *p* = 0.008, and between R1 and R3 (*p* = 0.021), R4 (*p* = 0.007), and R5 (*p* = .008). All other comparisons between stages did not show significant differences (*p* > 0.05).

#### 3.1.2 Average number of errors

When examining the average number of errors across the different learning stages (Figure 6B), a clear trend of decreasing errors also emerged: Acq. (M = 230, SD = 145.2), followed by a peak at R1 (M = 603.6, SD = 174), with subsequent reductions seen in R2 (M = 456.8, SD = 111), R3 (M = 341.7, SD = 183.2), R4 (M = 291.7, SD = 134.1), and R5 (M = 270.8, SD = 146.3). A Repeated Measures ANOVA, conducted without assuming sphericity (ε = 0.6932), showed significant differences in the average number of errors across learning stages, F(3.466, 27.73) = 10.49, *p* < 0.0001; *Post hoc* comparisons identified significant variations between Acq. and R1 (*p* = 0.001), and less pronounced yet significant differences between R1 and R3 (*p* = 0.008), R1 and R4 (*p* = 0.006), and R1 and R5 (*p* = 0.007). All other comparisons did not yield any significant differences between errors across different learning stages (*p* > 0.05).

#### 3.1.3 Type of error

We decided to conduct a focused analysis on errors during sessions where mice followed the response rule from the previous learning stage. Reversal 1 (R1) was selected as the primary stage for this examination due to its high incidence of response errors (Figure 6C).

Our approach to classifying perseverative versus learning errors was based on the methodology of Dickson et al. (2013), who used a 40% correct response cutoff to distinguish these error types. Errors in sessions with ≤ 40% correct responses were categorized as perseverative, reflecting adherence to the prior learning stage’s response rule, while errors in sessions with performance between 41 and 80% were classified as learning errors (Dickson et al., 2013).

However, in our study, behavior consistent with perseveration was observed even when performance exceeded 40%. This observation, derived from tracking behavioral performance across sessions, suggested that the 40% threshold underestimated perseverative behavior in our task. To address this, we established a cutoff of 45% correct responses to categorize the errors: those occurring in sessions with ≤ 45% correct responses were classified as perseverance errors, and errors in sessions with performance above 45% (46–100%) were classified as learning errors. We also extended the classification of learning errors to include sessions with performance above 80%, as these sessions often reflected behaviors consistent with gradual acquisition and refinement of the new response rule. Additionally, this classification accounts for errors made in sessions where mice adopted a “win-stay, lose-switch” strategy, which typically occur around 50% correct responses.

A paired *t*-test revealed no significant difference between perseveration errors (M = 277.9, SD = 120.9) and learning errors (M = 325.7, SD = 139.5) in the first reversal stage, despite a slight increase in learning errors, as observed in Figure 6C [t(8) = 0.7368, *p* = 0.4823].

A similar pattern was observed across subsequent reversals (R2–R5), although the total number of errors decreased compared to R1. In R2, a paired *t*-test revealed significantly fewer perseveration errors (M = 192.7, SD = 36.35) than learning errors [M = 264.1, SD = 87.91; t(8) = 2.821, p = 0.0225]. For R3, no significant difference was found between the two error types [perseveration: M = 152.2, SD = 144.0; learning: M = 189.4, SD = 62.19; t(8) = 0.8927, *p* = 0.3981]. In R4, perseveration (M = 99.00, SD = 67.92) remained significantly lower than learning errors [M = 192.7, SD = 97.46; t(8) = 2.777, *p* = 0.0240]. A similar result was observed in R5, with perseveration (M = 88.11, SD = 98.82) significantly lower than learning errors [M = 182.7, SD = 76.18; t(8) = 2.875, *p* = 0.0207].

### 3.2 Location discrimination task

All mice used in this study were able to learn the location discrimination serial-reversal task. This paradigm demonstrated faster acquisition and completion times compared to the visual discrimination task, though performance differences were observed between C57 and App^NL−G−F^mice. The C57 mice completed the task efficiently, averaging M = 8.5 days (SD = 0.52), with individual completion times ranging from 8 to 9 days. In contrast, App^NL−G−F^ mice required more time (M = 10.22 days, SD = 1.64) and displayed greater variability, with completion times ranging from 8 to 12 days.

#### 3.2.1 Average number of sessions

**FIGURE 7:**
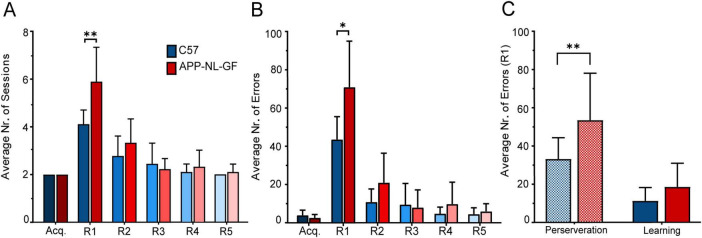
Performance in the location discrimination serial-reversal task. **(A)** Average number of sessions across all learning stages. **(B)** Average number of errors across all learning stages. **(C)** Comparison of perseverance errors and learning errors in R1. Mean (M) ± SD in each learning stage. Statistical significance indicated as *p < 0.05, **p < 0.001.

Even though there were individual as well as group differences in the amount of time necessary for the animals to complete the task, the general tendency was to converge toward the minimum number of sessions required to pass each stage - two consecutive sessions as seen in Figure 7A. A Two-Way Repeated Measures ANOVA indicated a significant interaction between Learning Stage and Genotype on the number of sessions to reach the passing criteria (≥ 80% correct responses in two consecutive sessions), F(5, 80) = 4.935, *p* < 0.001. Additionally, we found a significant main effect of Learning Stage, [F(2.994, 47.90) = 50.78, *p* < 0.001; ε = 0.5988], and Genotype [F(1, 16) = 7.806, *p* = 0.013]. No significant variability was attributed to individual differences among subjects, [F(16, 80) = 1.218, *p* = 0.273].

*Post hoc* comparisons revealed a significant difference between C57 and App^NL−G−F^mice in the first Reversal stage (R1), with C57 mice showing a mean (M) of 4.11 sessions (Standard Deviation, SD = 0.60) compared to App^NL−G−F^ mice (M = 5.88, SD = 1.453), *p* = 0.006. No significant differences were observed in other stages, including the Acquisition phase (Acq) where both C57 and APP mice completed the task with M = 2 sessions (SD = 0). Similarly, no significant differences were found in subsequent reversal stages: R2 (C57: M = 2.77, SD = 0.83; APP: M = 3.33, SD = 1), R3 (C57: M = 2.44, SD = 0.88; APP: M = 2.22, SD = 0.44), R4 (C57: M = 2.11, SD = 0.33; APP: M = 2.33, SD = 0.70), and R5 (C57: M = 2, SD = 0; APP: M = 2.11, SD = 0.33), all yielding *p* > 0.05.

Within-group analysis revealed distinct patterns of significant differences in the average number of sessions spent across learning stages for both C57 and App^NL−G−F^mice. For C57 mice, comparisons between R1 and all other stages, except R2, showed significant differences: R1 vs Acq. (*p* < 0.001), R1 vs. R3 (*p* = 0.017), R1 vs. R4 (*p* = 0.001), and R1 vs. R5 (*p* < 0.001). In contrast, the comparison between R1 and R2 only approached significance (*p* = 0.055), suggesting a less pronounced difference between these reversal stages.

In the App^NL−G−F^group, R1 showed significant differences when compared to all other learning stages, highlighting a consistent pattern: R1 vs. Acq. (*p* < 0.001), R1 vs. R2 (*p* = 0.008), R1 vs. R3 (*p* < 0.001), R1 vs. R4 (*p* = 0.001), and R1 vs. R5 (*p* = 0.001). Additionally, statistical analysis also identified significant differences between R2 and Acq. (*p* = 0.032), and between R2 and R5 (*p* = 0.043).

#### 3.2.2 Average number of errors

A similar trend was observed in terms of the average number of errors between C57 and App^NL−G−F^mice across the different learning stages (Figure 7B). A Two-way Repeated Measures ANOVA highlighted significant effects for the interaction between Learning Stage and Genotype, [F(5, 80) = 5.405, *p* < .001]. Significant main effects were observed for Learning Stage, [F(2.309, 36.95) = 75.72, *p* < .001; ε = 0.461], and for Genotype, [F(1, 16) = 7.037, *p* = 0.017]. Additionally, variability attributed to individual mice was also significant, [F(16, 80) = 1.803, *p* = 0.045]. The only statistically significant difference between groups, was once again observed in R1 (C57: M = 43.77, SD = 11.98; APP: M = 71.33, SD = 24.28; *p* = 0.010).

Conversely, the comparisons revealed no significant differences in the Acquisition stage (C57: M = 3.88, SD = 2.47; APP: M = 2.55, SD = 1.74, *p* = 0.206) R2 (C57: M = 10.88, SD = 6.86; APP: M = 21.11, SD = 15.22, *p* = 0.093) R3 (C57: M = 9.55, SD = 10.86; APP: M = 8, SD = 9.02, *p* = 0.746) R4 (C57: M = 4.66, SD = 3.27; APP: M = 9.77, SD = 11.23, *p* = 0.221) and R5 (C57: M = 4.44, SD = 3.12; APP: M = 5.88, SD = 3.75, *p* = 0.389).

Within group comparisons showed once again, differences between R1 and every other learning stage for control mice (R1 vs. Acq.: *p* < 0.001; R1 vs. R2: *p* < 0.001; R1 vs. R3: *p* < 0.001; R1 vs. R4: *p* < 0.001; R1 vs. R5: *p* < 0.001), whereas for App^NL−G−F^mice differences were found between Acq. and R2 (*p* = 0.031), and R1 versus the remaining learning stages (R1 vs. Acq.: *p* < 0.001; R1 vs. R2: *p* = 0.001; R1 vs. R3: *p* < 0.001; R1 vs. R4: *p* = 0.002; R1 vs. R5: *p* < 0.001).

#### 3.2.3 Type of error

To further investigate error types, we focused our analysis on R1 (Figure 7C), where the incidence of errors was highest. Subsequent reversals were excluded from analysis, as perseverative behavior was limited to just three App^NL−G−F^ mice, each displaying it in a single session during R2, with one of these mice also demonstrating perseveration in a single session during R3.

A Mixed-Effects Model (REML) revealed no significant interaction between Error Type and Genotype [F(1, 32) = 1.667, *p* = 0.206]. However, significant main effects were observed for both Error Type [F(1, 32) = 31.14, *p* < 0.001] and individual mice [F(1, 32) = 7.362, *p* = 0.011], indicating that variability across individual mice contributed significantly to the model. *Post hoc* analysis using Fisher’s LSD revealed a statistically significant difference in perseverative errors between C57 and App^NL−G−F^ groups (C57: M = 32.78, SD = 11.13; APP: M = 53.11, SD = 24.55; *p* = 0.008), but not in learning errors (C57: M = 11.00, SD = 6.946; APP: M = 18.22, SD = 12.35; *p* = 0.322). Statistically significant within-group differences in error type were also observed in both groups (C57: *p* = 0.005; APP: *p* < 0.001).

## 4 Discussion

### 4.1 Behavioral tasks

In the visual discrimination task, the C57BL/6 mice displayed a trend in the average number of errors across learning stages, which was also reflected in the average number of sessions, revealing a progressive improvement in performance over time. The lack of significant differences between R1 and R2, and among subsequent reversal stages, suggests that despite the initial struggle mice gradually adapt to the new reward contingencies. And although there was a slight increase in the average number of learning errors in comparison with perseveration errors, the difference was not statistically significant.

The App^NL−G−F^ mice were not included in the visual discrimination serial-reversal task due to its inherent difficulty, even for C57 mice. Tasks requiring multiple reversals are cognitively demanding, as they rely heavily on cognitive flexibility. Given the progressive cognitive decline in App^NL−G−F^ mice by 8–10 months, we anticipated that the pathology would severely interfere with task completion (Mehla et al., 2019). Touchscreen paradigms are highly sensitive to subtle cognitive impairments, as early-stage App^NL−G−F^ mice (4–6 months) have been shown to complete simple visual discrimination tasks but perform poorly on more demanding paired-associate learning and location-based tasks. Indeed, Van den Broeck et al. (2019) reported that in APPPS1-21 mice, cognitive flexibility impairments emerge at early stages of pathology, with transgenic animals requiring more sessions than controls to complete a single reversal (Van den Broeck et al., 2019). Given these challenges, we did not expect App^NL−G−F^ mice to consistently complete tasks with 5 reversals, as most would likely fail beyond the first reversal.

On the other hand, in the location discrimination serial-reversal task, despite the considerable gap in terms of both the average number of sessions and average number of errors across the different learning stages, both wild-type and App^NL−G−F^mice showed a clear progression toward asymptotic performance. Mirroring the performance of the C57 mice in the visual discrimination task, both groups experienced significant challenges when first adjusting to reversed reward contingencies, reflecting the difficulty in overriding previously learned associations.

Both C57 and App^NL−G−F^ mice revealed significant differences in terms of both average number of sessions and errors, particularly in R1. This suggests a stark contrast in cognitive flexibility between genotypes and better adaptability, with C57 mice adjusting more quickly to the reversals and making fewer mistakes compared to the App^NL−G−F^ cohort. Furthermore, when examining the specific type of errors (perseverative versus learning errors), significant differences emerged between the genotypes, with App^NL−G−F^ mice generally committing more perseverative errors. These findings underscore potential genotype-specific challenges in shifting strategies after rule changes, and overall cognitive flexibility, which could reflect broader implications in neurological or cognitive research, particularly in understanding conditions such as Alzheimer’s disease (Braak and Braak, 1991; Allegri et al., 2000; Llinas and Moreno, 2017; Walker et al., 2018; Guarino et al., 2019; McAllister et al., 2020; Knopman et al., 2021; Sasaguri et al., 2022).

The accessibility of the location discrimination task was essential for enabling App^NL−G−F^ mice to perform a serial-reversal learning paradigm, given the amyloid burden, gliosis, and cholinergic deficits reported in 8–10 months-old animals (Shah et al., 2018; Latif-Hernandez et al., 2019, 2020; Mehla et al., 2019). These cognitive impairments align with the findings of Sutoko et al. (2021), who utilized machine learning methods to identify preclinical AD risk in App^NL−G−F^ mice, highlighting significant behavioral changes within the 8–12 months age window, when cognitive symptoms become increasingly apparent (Sutoko et al., 2021). These impairments reflect the early vulnerability of hippocampal circuits, which are central to the location-discrimination task (Ettcheto et al., 2018; McAllister et al., 2020; Knopman et al., 2021)

The discrepancies observed in these tasks might stem from the extended time needed to establish and reverse the association between specific visual inputs, such as virtual objects or images, and a reward. Although the number of sessions required for the animals to learn the new reward contingency in the visual discrimination task decreased over time, perseverative behavior persisted until the fifth reversal. In contrast, in the location discrimination task, such behavior was mostly observed in R1, and rarely displayed in subsequent reversals.. By the fourth reversal, almost all mice had reached a performance asymptote, typically requiring just two sessions to meet the passing criterion.

Our findings suggest that further research is needed to fully understand the behavioral dynamics between these two tasks. Our version of the location discrimination task, differing from those reported in previous studies by employing “across session” instead of “within-session” reversals, presents unique challenges in terms of overwriting the previously acquired rules. This is not only due to the considerable number of individual trials required to meet the passing criterion, which strengthens the association between the rules and outcomes, but also due to the presence of distractor images that could influence decision-making. Interestingly, animals in the location discrimination task tended to ignore the visual cues and consistently choose a specific side, suggesting that in this context, visual stimuli do not significantly impact their behavior.

One possible explanation for this behavior is the evolutionary bias of rodents toward spatial strategies, which are vital for survival behaviors like foraging, burrowing, and predator avoidance, using landmarks and shortcuts to minimize predation risks (Shettleworth, 2009; Wang et al., 2021; Khalil, 2024; Lai et al., 2024). This evolutionary bias seems to reflect the rodent brain’s specialization for spatial processing, a capability that is further enhanced by repeated exposure to spatial tasks. The location discrimination task may engage brain regions involved in spatial navigation, such as the hippocampal formation and cortical areas including the entorhinal and retrosplenial cortex, which are known to encode spatial maps and navigation metrics, facilitating efficient adaptation to spatial environments (O’Keefe and Dostrovsky, 1971; Hafting et al., 2005; McNaughton et al., 2006; Keene et al., 2016; Esteves et al., 2021).

Conversely, the visual discrimination task, in addition to the different visual processing regions essential for image or object recognition, requires abstract stimulus-response associations and reversal learning, which primarily rely on the prefrontal cortex (PFC) for cognitive flexibility and the striatum for reinforcement learning (Schultz, 1998; Birrell and Brown, 2000; Brigman et al., 2010; Euston et al., 2012; Marquardt et al., 2017; Biró et al., 2019; Piantadosi et al., 2019; Uddin, 2021). Sensory constraints such as poor visual acuity and dichromatic vision, may further hinder rodents’ ability to process visual stimuli, adding to the challenges of abstract visual tasks (Huberman and Niell, 2011; Abballe and Asari, 2022).

These challenges align with findings from dual-cue paradigms, where rodents initially rely on hippocampus-dependent spatial strategies, but transition to striatum-based response learning after extended training or when the hippocampus is impaired (Packard and McGaugh, 1996). This shift underscores the distinct neural systems underpinning cognitive flexibility and learning strategies, particularly in tasks requiring adaptations to changing rules or environments.

Lastly, it is also important to acknowledge the length of the training procedures, which can be quite onerous for both the animals and the experimenters. This was particularly evident in the visual discrimination serial reversal task, with some animals taking up to 4 months to complete the task. The lengthy nature of this experiment was also noticeable during the later reversals, when some mice began to lose motivation, which can lead to suboptimal performance levels.

To reduce training times, one option could be to limit the serial-reversal task to four reversals, as performance improvements were evident after the initial reversals. While this five-reversal scheme provided a clear marker for when learning stabilized across reversals, using alternative touchscreen paradigms that are less time-intensive may offer practical advantages. The classic visual discrimination task, although widely used, is rarely applied in a serial-reversal format, and its prolonged duration may limit its feasibility. Simpler tasks, fewer reversals or alternative cognitive demands could provide more efficient options for assessing cognitive flexibility while reducing the experimental timeline.

### 4.2 Touchscreen apparatus

One of the most important aspects of any scientific endeavor is exploration, and while it is crucial to standardize behavioral procedures in research, allowing for experimentation and the expansion of methods is equally vital. This requires different labs experimenting with various hardware and software configurations for a comprehensive assessment of cognitive functions, as it is important to determine whether certain elements or steps in behavioral tasks, especially in touchscreen tasks, are indispensable features, or if they are subject to improvement or even elimination.

Our group tested various configurations before adopting a design inspired by the original touchscreen chambers, however, other groups have introduced their own designs without significantly deviating from the outcomes observed with standard setups (Pineño, 2014; O’Leary et al., 2018; Wiesbrock et al., 2022; Eleftheriou et al., 2023).

Among the various configurations we tested, selecting an appropriate touchscreen was critical. Many commercially available touchscreens designed for Raspberry Pi devices that we tested, were ultimately unsuitable for our purposes, as they often failed to register rodent touch input accurately. This hindered the animals’ ability to associate specific actions with outcomes, rendering these devices impractical for our tasks. As a result, we adopted the Samsung SM-T350 tablet, which provided a good touch sensitivity, compatibility with an Android platform, and greater flexibility in task development. This choice allowed us to create a library of images and virtual objects, including both color and black and white options, to accommodate the specific requirements of different experiments.

Regarding task parameters, we found that using a fixed 5 s inter-stimulus interval (ITI), set to allow images to appear while the animal approached the reward, was effective. This approach differs from standard setups, where the ITI begins after reward collection, but provided clear visibility for mice without requiring them to initiate each trial (Horner et al., 2013; Mar et al., 2013). We also excluded correction trials from the standard touchscreen setup. Although, our user interface includes the option for correction trials, pilot testing revealed that they did not enhance animal performance or reduce the time required for each learning stage.

To summarize, our custom-built Android-based touchscreen system is scalable, modular, and flexible, allowing simultaneous use of multiple units while maintaining standardization across experiments. Its flexibility enables task-customization for a wide range of cognitive tasks, enabling the evaluation of disease progression and treatment efficacy, as well as for testing diverse mouse models, such as transgenic strains used in studies of neurodegenerative diseases. The system’s modularity also facilitates adaptation for specific experimental goals, such as pairing touchscreen tasks with electrophysiology or imaging techniques, and its scalability and cost-effectiveness make it accessible for longitudinal studies and large-scale pharmacological screening (Dumont et al., 2021).

Additionally, exploring new research avenues, such as integrating touchscreen technology directly into animals’ home cages, holds promise for significant advancements (Singh et al., 2019; Kahnau et al., 2023; Hsieh et al., 2024). This strategy could not only mitigate stress from exposure to unfamiliar environments but also substantially reduce human-animal interaction, therefore minimizing the introduction of confounding variables that could skew results despite the standardization of experimental protocols. Allowing for the assessment of ethologically relevant behavior, while virtually eliminating experimenter involvement could represent a step forward in creating more humane and precise behavioral research methodologies.

## 5 Conclusion

Our custom-built touchscreen apparatus for mice has proven to be both practical and cost-effective, offering a viable alternative to more expensive commercial systems. By leveraging commercially available computer tablets integrated with a Raspberry Pi, our system not only reduces equipment costs but also provides detailed insights into cognitive flexibility and behavioral strategies. Through this approach, we developed both visual discrimination and location discrimination tasks with five reversals each, which allowed us to observe distinct performance patterns. Despite similarities in their overall design, the two tasks require varying levels of cognitive flexibility, underscoring the need for further research into the specific mechanisms underlying these differences, and their implications for understanding cognitive and behavioral processes in different mouse models, and a broader comprehension of both normal and pathological brain functions.

## Data Availability

The datasets presented in this study can be found in online repositories. The names of the repository/repositories and accession number(s) can be found below: https://github.com/Mohajerani-Lab/touchscreen-chamber-unity.

## References

[B1] Abballe L. Asari H. (2022). Natural image statistics for mouse vision. *PLoS One* 17:e0262763. 10.1371/journal.pone.0262763 35051230 PMC8775586

[B2] Allegri R. F. Harris P. Drake M. (2000). La evaluación neuropsicológica en la Enfermedad de Alzheimer. *Rev. Neurol. Arg.* 1431 11–15.

[B3] Alsiö J. Nilsson S. Gastambide F. Wang R. Dam S. Mar A. (2015). The role of 5-HT2C receptors in touchscreen visual reversal learning in the rat: A cross-site study. *Psychopharmacology* 232 4017–4031. 10.1007/s00213-015-3963-5 26007324 PMC4600472

[B4] Barnard I. L. Onofrychuk T. McElroy D. Howland J. (2021). The Touchscreen-based trial-unique, nonmatching-to-location (TUNL) task as a measure of working memory and pattern separation in rats and mice. *Curr. Protoc.* 1:e238. 10.1002/cpz1.238 34570962

[B5] Birrell J. M. Brown V. J. (2000). Medial frontal cortex mediates perceptual attentional set shifting in the rat. *J. Neurosci*. 20:43204324. 10.1523/JNEUROSCI.20-11-04320.2000 10818167 PMC6772641

[B6] Biró S. Lasztóczi B. Klausberger T. (2019). A visual two-choice rule-switch task for head-fixed mice. *Front. Behav. Neurosci*. 13:119. 10.3389/fnbeh.2019.00119 31244622 PMC6562896

[B7] Bloom G. S. (2014). Amyloid-β and tau: The trigger and bullet in Alzheimer disease pathogenesis. *JAMA Neurol.* 71 505–508. 10.1001/jamaneurol.2013.5847 24493463 PMC12908160

[B8] Boulougouris V. Dalley J. W. Robbins T. W. (2007). Effects of orbitofrontal, infralimbic and prelimbic cortical lesions on serial spatial reversal learning in the rat. *Behav. Brain Res.* 179 219–228. 10.1016/j.bbr.2007.02.005 17337305

[B9] Braak H. Braak E. (1991). Neuropathological stageing of Alzheimer-related changes. *Acta Neuropathol.* 82 239–259. 10.1007/BF00308809 1759558

[B10] Brigman J. L. Graybeal C. Holmes A. (2010). Predictably irrational: Assaying cognitive inflexibility in mouse models of schizophrenia. *Front. Neurosci.* 4:13. 10.3389/neuro.01.013.2010 20859447 PMC2938983

[B11] Bryce C. A. Howland J. G. (2015). Stress facilitates late reversal learning using a touchscreen-based visual discrimination procedure in male long evans rats. *Behav. Brain Res.* 278 21–28. 10.1016/j.bbr.2014.09.027 25251839 PMC4457515

[B12] Bussey T. J. Everitt B. J. Robbins T. W. (1997). Dissociable effects of cingulate and medial frontal cortex lesions on stimulus-reward learning using a novel pavlovian autoshaping procedure for the rat: Implications for the neurobiology of emotion. *Behav. Neurosci.* 111 908–919. 10.1037/0735-7044.111.5.908 9383513

[B13] Bussey T. J. Muir J. L. Robbins T. W. (1994). A novel automated touchscreen procedure for assessing learning in the rat using computer graphic stimuli. *Neurosci. Res. Commun.* 15 103–110.

[B14] Bussey T. J. Padain T. Skillings E. Winters B. Morton A. Saksida L. (2008). The touchscreen cognitive testing method for rodents: How to get the best out of your rat. *Learn. Mem.* 15 516–523. 10.1101/lm.987808 18612068 PMC2505319

[B15] Bussey T. J. Saksida L. M. Rothblat L. A. (2001). Discrimination of computer-graphic stimuli by mice: A method for the behavioral characterization of transgenic and gene-knockout models. *Behav. Neurosci.* 115 957–960. 10.1037/0735-7044.115.4.957 11508736

[B16] Castañé Anna A. Theobald D. E. H. Robbins T. W. (2010). Selective lesions of the dorsomedial striatum impair serial spatial reversal learning in rats. *Behav. Brain Res.* 210 74–83. 10.1016/j.bbr.2010.02.017 20153781 PMC3038258

[B17] Chudasama Y. Robbins T. W. (2003). Behavioral/systems/cognitive dissociable contributions of the orbitofrontal and infralimbic cortex to pavlovian autoshaping and discrimination reversal learning: Further evidence for the functional heterogeneity of the rodent frontal cortex. *J. Neurosci.* 23 8771–8780. 14507977 10.1523/JNEUROSCI.23-25-08771.2003 PMC6740430

[B18] Clatworthy P. L. Lewis S. Brichard L. Hong Y. Izquierdo D. Clark L. (2009). Dopamine release in dissociable striatal subregions predicts the different effects of oral methylphenidate on reversal learning and spatial working memory. *J. Neurosci.* 29 4690–4696. 10.1523/JNEUROSCI.3266-08.2009 19369539 PMC6665353

[B19] Cools R. Clark L. Owen A. Robbins T. (2002). Defining the neural mechanisms of probabilistic reversal learning using event-related functional magnetic resonance imaging. *J. Neurosci.* 22 4563–4567. 12040063 10.1523/JNEUROSCI.22-11-04563.2002 PMC6758810

[B20] Creighton S. D. Collett H. Zonneveld P. Pandit R. Huff A. Jardine K. (2019). Development of an “object category recognition” task for mice: Involvement of muscarinic acetylcholine receptors. *Behav. Neurosci.* 133 527–536. 10.1037/bne0000331 31246078

[B21] D’Cruz A. M. Ragozzino M. Mosconi M. Shrestha S. Cook E. Sweeney J. (2013). Reduced behavioral flexibility in autism spectrum disorders. *Neuropsychology* 27 152–160. 10.1037/a0031721 23527643 PMC3740947

[B22] Dias R. Robbins T. W. Roberts A. C. (1996). Dissociation in prefrontal cortex of affective and attentional shifts. *Nature* 380 69–72. 10.1038/380069a0 8598908

[B23] Dickson P. E. Corkill B. McKimm E. Miller M. Calton M. Goldowitz D. (2013). Effects of stimulus salience on touchscreen serial reversal learning in a mouse model of fragile X syndrome. *Behav. Brain Res.* 252 126–135. 10.1016/j.bbr.2013.05.060 23747611 PMC3854797

[B24] Dumont J. R. Salewski R. Beraldo F. (2021). Critical mass: The rise of a touchscreen technology community for rodent cognitive testing. *Genes Brain Behav.* 20:e12650. 10.1111/gbb.12650 32141694

[B25] Eleftheriou C. Clarke T. Poon V. Zechner M. Duguid I. (2023). Visiomode: An open-source platform for building rodent touchscreen-based behavioral assays. *J. Neurosci. Methods* 386:109779. 10.1016/j.jneumeth.2022.109779 36621552 PMC10375831

[B26] Esteves I. M. Chang H. Neumann A. Sun J. Mohajerani M. McNaughton B. (2021). Spatial information encoding across multiple neocortical regions depends on an intact hippocampus. *J. Neurosci.* 41 307–319. 10.1523/JNEUROSCI.1788-20.2020 33203745 PMC7810652

[B27] Ettcheto M. Abad S. Petrov D. Pedrós I. Busquets O. Sánchez-López E. (2018). Early preclinical changes in hippocampal CREB-binding protein expression in a mouse model of familial Alzheimer’s disease. *Mol. Neurobiol.* 55 4885–4895. 10.1007/s12035-017-0690-4 28752224

[B28] Euston D. R. Gruber A. J. McNaughton B. L. (2012). The role of medial prefrontal cortex in memory and decision making. *Neuron* 76:10571070. 10.1016/j.neuron.2012.12.002 23259943 PMC3562704

[B29] Ferster C. B. (1953). The use of the free operant in the analysis of behavior. *Psychol. Bull.* 50 189–208. 10.1037/h0055514 13074442

[B30] Folch J. Ettcheto M. Petrov D. Abad S. Pedrós I. Marin M. (2018). Review of the advances in treatment for Alzheimer disease: Strategies for combating β-amyloid protein. *Neurología (English Edition)* 33 47–58. 10.1016/j.nrleng.2015.03.019 25976937

[B31] Fowler K. (1980). Object discrimination by rats: The role of frontal and hippocampal systems in retention and reversal. *Physiol. Behav.* 24 33–38. 7384248 10.1016/0031-9384(80)90010-4

[B32] Graybeal C. Feyder M. Schulman E. Saksida L. Bussey T. Brigman J. (2011). Paradoxical reversal learning enhancement by stress or prefrontal cortical damage: Rescue with BDNF. *Nat. Neurosci.* 14 1507–1509. 10.1038/nn.2954 22057192 PMC3389817

[B33] Gruner P. Pittenger C. (2017). Cognitive inflexibility in obsessive-compulsive disorder. *Neuroscience* 345 243–255. 10.1016/j.neuroscience.2016.07.030 27491478 PMC5288350

[B34] Guardia-Laguarta C. Pera M. Clarimón J. Molinuevo J. Sánchez-Valle R. Lladó A. (2010). Clinical, neuropathologic, and biochemical profile of the amyloid precursor protein I716F mutation. *J. Neuropathol. Exp. Neurol.* 69 53–59. 10.1097/NEN.0b013e3181c6b84d 20010303

[B35] Guarino A. Favieri F. Boncompagni I. Agostini F. Cantone M. Casagrande M. (2019). Executive functions in Alzheimer disease: A systematic review. *Front. Aging Neurosci*. 10:437. 10.3389/fnagi.2018.00437 30697157 PMC6341024

[B36] Hafting T. Fyhn M. Molden S. Moser M. Moser E. (2005). Microstructure of a spatial map in the entorhinal cortex. *Nature* 436 801–806. 10.1038/nature03721 15965463

[B37] Hampshire A. Owen A. M. (2006). Fractionating attentional control using event-related fMRI. *Cereb. Cortex* 16 1679–1689. 10.1093/cercor/bhj116 16436686

[B38] Harper J. D. Lansbury P. T. (1997). Models of amyloid seeding in Alzheimer’s disease and scrapie: Mechanistic truths and physiological consequences of the time-dependent solubility of amyloid proteins. *Annu. Rev. Biochem*. 66 385–407. 10.1146/annurev.biochem.66.1.385 9242912

[B39] Hornak J. O’Doherty J. Bramham J. Rolls E. Morris R. Bullock P. (2004). Reward-related reversal learning after surgical excisions in orbito-frontal or dorsolateral prefrontal cortex in humans. *J. Cogn. Neurosci.* 16 463–478. 15072681 10.1162/089892904322926791

[B40] Horner A. E. Heath C. Hvoslef-Eide M. Kent B. Kim C. Nilsson S. (2013). The touchscreen operant platform for testing learning and memory in rats and mice. *Nat. Protoc.* 8 1961–1984. 10.1038/nprot.2013.122 24051959 PMC3914026

[B41] Hsieh C. M. Hsu C. Chen J. Liao L. (2024). AI-powered home cage system for real-time tracking and analysis of rodent behavior. *iScience* 27:111223. 10.1016/j.isci.2024.111223 39605925 PMC11600061

[B42] Huberman A. D. Niell C. M. (2011). What can mice tell us about how vision works? *Trends Neurosci.* 34 464–473. 10.1016/j.tins.2011.07.002 21840069 PMC3371366

[B43] Hvoslef-Eide M. Mar A. Nilsson S. Alsiö J. Heath C. Saksida L. (2015). The NEWMEDS rodent touchscreen test battery for cognition relevant to schizophrenia. *Psychopharmacology* 232 3853–3872. 10.1007/s00213-015-4007-x 26202612

[B44] Hvoslef-Eide M. Nilsson S. Saksida L. Bussey T. (2016). Cognitive translation using the rodent touchscreen testing approach. *Curr. Top. Behav. Neurosci*. 28 423–447. 10.1007/7854_2015_5007 27305921

[B45] Izquierdo A. Jentsch J. D. (2012). Reversal learning as a measure of impulsive and compulsive behavior in addictions. *Psychopharmacology* 219 607–620. 10.1007/s00213-011-2579-7 22134477 PMC3249486

[B46] Izquierdo A. Brigman J. L. Radke A. K. Rudebeck P. Holmes A. (2017). The neural basis of reversal learning: An updated perspective. *Neuroscience* 345 12–26. 10.1016/j.neuroscience.2016.03.021 26979052 PMC5018909

[B47] Izquierdo A. Wiedholz L. Millstein R. Yang R. Bussey T. Saksida L. (2006). Genetic and dopaminergic modulation of reversal learning in a touchscreen-based operant procedure for mice. *Behav. Brain Res.* 171 181–188. 10.1016/j.bbr.2006.03.029 16713639

[B48] Jara-Rizzo M. F. Navas J. Rodas J. Perales J. (2020). Decision-making inflexibility in a reversal learning task is associated with severity of problem gambling symptoms but not with a diagnosis of substance use disorder. *BMC Psychol.* 8:120. 10.1186/s40359-020-00482-6 33168098 PMC7654010

[B49] Kahnau P. Mieske P. Wilzopolski J. Kalliokoski O. Mandillo S. Holter S. (2023). A systematic review of the development and application of home cage monitoring in laboratory mice and rats. *BMC Biol.* 21:256. 10.1186/s12915-023-01751-7 37953247 PMC10642068

[B50] Keene C. S. Bladon J. McKenzie S. Liu C. O’Keefe J. Eichenbaum H. (2016). Complementary functional organization of neuronal activity patterns in the perirhinal, lateral entorhinal, and medial entorhinal cortices. *J. Neurosci.* 36 3660–3675. 10.1523/JNEUROSCI.4368-15.2016 27030753 PMC4812128

[B51] Khalil M. H. (2024). Environmental enrichment: A systematic review on the effect of a changing spatial complexity on hippocampal neurogenesis and plasticity in rodents, with considerations for translation to urban and built environments for humans. *Front. Neurosci*. 18:1368411. 10.3389/fnins.2024.1368411 38919908 PMC11196820

[B52] Kim C. H. Romberg C. Hvoslef-Eide M. Oomen C. Mar A. Heath C. (2015). Trial-unique, delayed nonmatching-to-location (TUNL) touchscreen testing for mice: Sensitivity to dorsal hippocampal dysfunction. *Psychopharmacology* 232 3935–3945. 10.1007/s00213-015-4017-8 26173611 PMC4600470

[B53] Kim M. Kwak C. Yu N. Kanng B. (2016). Optimization of the touchscreen paired-associate learning (PAL) task for mice and its dorsal hippocampal dependency. *Anim. Cells Syst.* 20 229–236. 10.1080/19768354.2016.1221855

[B54] Knopman D. S. Amieva H. Petersen R. Chételat G. Holtzman D. Hyman B. (2021). Alzheimer disease. *Nat. Rev. Dis. Primers* 7:33. 10.1038/s41572-021-00269-y 33986301 PMC8574196

[B55] Kosaki Y. Watanabe S. (2012). Dissociable roles of the medial prefrontal cortex, the anterior cingulate cortex, and the hippocampus in behavioural flexibility revealed by serial reversal of three-choice discrimination in rats. *Behav. Brain Res.* 227 81–90. 10.1016/j.bbr.2011.10.039 22061799

[B56] Kwak C. Lim C. S. Kaang B. K. (2016). Assessments of cognitive abilities in a mouse model of Parkinson’s disease with a touch screen test. *Behav. Brain Res.* 301 63–71. 10.1016/j.bbr.2015.12.016 26698399

[B57] Kwak C. Lim C.-S. Kaang B.-K. (2015). Development of a touch-screen-based paradigm for assessing working memory in the mouse. *Exp. Neurobiol.* 24 84–89. 10.5607/en.2015.24.1.84 25792872 PMC4363337

[B58] Lacoursiere S. G. Safar J. Westaway D. Mohajerani M. Sutherland R. (2022). The effect of Aβ seeding is dependent on the presence of knock-in genes in the AppNL-G-F mice. *Front. Dement.* 1:941879. 10.3389/frdem.2022.941879 39081481 PMC11285652

[B59] Lafleche G. Albert M. S. (1995). Executive function deficits in mild Alzheimer’s disease. *Neuropsychology* 9 313–320.

[B60] Lai A. T. Espinosa G. Wink G. Angeloni C. Dombeck A. Maclver M. (2024). A robot-rodent interaction arena with adjustable spatial complexity for ethologically relevant behavioral studies. *Cell Rep.* 43:113671. 10.1016/j.celrep.2023.113671 38280195

[B61] Latif-Hernandez A. Sabanov V. Ahmed T. Craessaerts K. Saito T. Saido T. (2020). The two faces of synaptic failure in App NL-G-Fknock-in mice. *Alzheimer’s Res. Therapy* 12 100. 10.1186/s13195-020-00667-6 32838792 PMC7445922

[B62] Latif-Hernandez A. Shah D. Craessaerts K. Saido T. Saito T. De Strooper B. (2019). Subtle behavioral changes and increased prefrontal-hippocampal network synchronicity in APP NL-G-F mice before prominent plaque deposition. *Behav. Brain Res.* 364 431–441. 10.1016/j.bbr.2017.11.017 29158112

[B63] Llinas R. Moreno H. (2017). Perspective on calcium and Alzheimer disease. *Alzheimer’s Dement.* 13 1–2. 10.1016/j.jalz.2017.01.004 28130964

[B64] Lucantonio F. Caprioli D. Schoenbaum G. (2014). Transition from “model-based” to “model-free” behavioral control in addiction: Involvement of the orbitofrontal cortex and dorsolateral striatum. *Neuropharmacology* 76 407–415. 10.1016/j.neuropharm.2013.05.033 23752095 PMC3809026

[B65] Mar A. C. Horner A. Nilsson S. Alsiö J. Kent B. Kim C. (2013). The touchscreen operant platform for assessing executive function in rats and mice. *Nat. Protoc.* 8 1985–2005. 10.1038/nprot.2013.123 24051960 PMC4131754

[B66] Marazziti D. Consoli G. Picchetti M. Carlini M. Faravelli L. (2010). Cognitive impairment in major depression. *Eur. J. Pharmacol.* 626 83–86. 10.1016/j.ejphar.2009.08.046 19835870

[B67] Markham K. R. Butt A. E. Dougher M. J. (1996). A computer touch-screen apparatus for training visual discriminations in rats. *Training* 65 173–182. 10.1901/jeab.1996.65-173 8583196 PMC1350070

[B68] Marquardt K. Sigdel R. Brigman J. L. (2017). Touch-screen visual reversal learning is mediated by value encoding and signal propagation in the orbitofrontal cortex. *Neurobiol. Learn. Mem.* 139 179–188. 10.1016/j.nlm.2017.01.006 28111339 PMC5372695

[B69] McAllister B. B. Lacoursiere S. Sutherland R. Mohajerani M. (2020). Intracerebral seeding of amyloid-β and tau pathology in mice: Factors underlying prion-like spreading and comparisons with α-synuclein. *Neurosci. Biobehav. Rev*. 112 1–27. 10.1016/j.neubiorev.2020.01.026 31996301

[B70] McNaughton B. L. Battaglia F. Jensen O. Moser E. Moser M. (2006). Path integration and the neural basis of the “cognitive map”. *Nat. Rev. Neurosci.* 7 663–678. 10.1038/nrn1932 16858394

[B71] Mehla J. Deibel S. Karem H. Hong N. Hossain S. Lacoursiere S. (2023). Repeated multi-domain cognitive training prevents cognitive decline, anxiety and amyloid pathology found in a mouse model of Alzheimer disease. *Commun. Biol.* 6: 1145. 10.1038/s42003-023-05506-6 37950055 PMC10638434

[B72] Mehla J. Lacoursiere S. Lapointe V. McNaughton B. Sutherland R. McDonald R. (2019). Age-dependent behavioral and biochemical characterization of single APP knock-in mouse (APPNL-G-F/NL-G-F) model of Alzheimer’s disease. *Neurobiol. Aging* 75 25–37. 10.1016/j.neurobiolaging.2018.10.026 30508733

[B73] Millan M. J. Agid Y. Brüne M. Bullmore E. Carter C. Clayton N. (2012). Cognitive dysfunction in psychiatric disorders: Characteristics, causes and the quest for improved therapy. *Nat. Rev. Drug Discov.* 11 141–168. 10.1038/nrd3628 22293568

[B74] Monni A. Scandola M. Hélie S. Scalas L. (2023). Cognitive flexibility assessment with a new reversal learning task paradigm compared with the Wisconsin card sorting test exploring the moderating effect of gender and stress. *Psychol. Res.* 87 1439–1453. 10.1007/s00426-022-01763-y 36369387 PMC9651887

[B75] Nilsson P. Saito T. Saido T. C. (2014). New mouse model of Alzheimer’s. *ACS Chem. Neurosci.* 5 499–502. 10.1021/cn500105p 24852598 PMC4102956

[B76] Nilsson S. R. O. Saksida L. M. Bussey T. J. (2016). *Cognitive Translation Using the Rodent Touchscreen Testing Approach.* Berlin: Springer, 423–447. 10.1007/7854 27305921

[B77] Nithianantharajah J. McKechanie A. Stewart T. Johnstone M. Blackwood D. St Clair D. (2015). Bridging the translational divide: Identical cognitive touchscreen testing in mice and humans carrying mutations in a disease- relevant homologous gene. *Sci. Rep.* 5 3–7. 10.1038/srep14613 26423861 PMC4589696

[B78] O’Keefe J. M. Dostrovsky J. O. (1971). The hippocampus as a spatial map. Preliminary evidence from unit activity in the freely-moving rat. *Brain Res.* 34 171–175. 10.1016/0006-8993(71)90358-1 5124915

[B79] O’Leary J. D. O’Leary E. Cryan F. Nolan Y. (2018). A low-cost touchscreen operant chamber using a Raspberry Pi™. *Behav. Res. Methods* 50 2523–2530. 10.3758/s13428-018-1030-y 29520633

[B80] Odland A. U. Sandahl R. Andreasen J. T. (2021). Sequential reversal learning: A new touchscreen schedule for assessing cognitive flexibility in mice. *Psychopharmacology* 238 383–397. 10.1007/s00213-020 33123820

[B81] Packard M. G. McGaugh J. L. (1996). Inactivation of hippocampus or caudate nucleus with lidocaine differentially affects expression of place and response learning. *Neurobiol. Learn. Mem.* 65 65–72. 10.1006/nlme.1996.0007 8673408

[B82] Phillips B. U. Heath C. Ossowska Z. Bussey T. Saksida L. (2017). Optimisation of cognitive performance in rodent operant (touchscreen) testing: Evaluation and effects of reinforcer strength. *Learn Behav.* 45 252–262. 10.3758/s13420-017-0260-7 28205186 PMC5565648

[B83] Piantadosi P. T. Lieberman A. G. Pickens C. L. Bergstrom H. C. Holmes A. (2019). A novel multichoice touchscreen paradigm for assessing cognitive flexibility in mice. *Learn. Mem*. 26:2430. 10.1101/lm.048264.118 30559117 PMC6298539

[B84] Pineño O. (2014). ArduiPod box: A low-cost and open-source Skinner box using an iPod Touch and an Arduino microcontroller. *Behav. Res. Methods* 46 196–205. 10.3758/s13428-013-0367-5 23813238

[B85] Pinkston J. W. (2022). Operant responding: Beyond rate and interresponse times. *Brain Res. Bull.* 186 79–87. 10.1016/j.brainresbull.2022.05.009 35644432

[B86] Saifullah M. A. Komine O. Dong Y. Fukumoto K. Sobue A. Endo F. (2020). Touchscreen-based location discrimination and paired associate learning tasks detect cognitive impairment at an early stage in an App knock-in mouse model of Alzheimer’s disease. *Mol. Brain* 13:141. 10.1186/s13041-020-00690-6 33183323 PMC7664057

[B87] Saito T. Matsuba Y. Mihira N. Takano J. Nilsson P. Itohara S. (2014). Single App knock-in mouse models of Alzheimer’s disease. *Nat. Neurosci.* 17 661–663. 10.1038/nn.3697 24728269

[B88] Sakagami T. Lattal K. A. (2016). The other shoe: An early operant conditioning chamber for pigeons. *Behav. Anal.* 39 25–39. 10.1007/s40614-016-0055-8 27606188 PMC4883506

[B89] Sasaguri H. Hashimoto S. Watamura N. Sato K. Takamura R. Nagata K. (2022). Recent advances in the modeling of Alzheimer’s disease. *Front. Neurosci*. 31:807473. 10.3389/fnins.2022.807473 35431779 PMC9009508

[B90] Schultz W. (1998). Predictive reward signal of dopamine neurons. *J. Neurophysiol*. 80:127. 10.1152/jn.1998.80.1.1 9658025

[B91] Shah D. Latif-Hernandez A. De Strooper B. Saito T. Saido T. Verhoye M. (2018). Spatial reversal learning defect coincides with hypersynchronous telencephalic BOLD functional connectivity in APPNL-F/NL-F knock-in mice. *Sci. Rep.* 8:664. 10.1038/s41598-018-24657-9 29674739 PMC5908850

[B92] Shettleworth S. J. (2009). *Cognition, Evolution, and Behavior*, 2nd Edn. New York: Oxford University Press.

[B93] Shin J.-Y. Yu S. Yu U. Jo S. Ahn J. (2010). Swedish mutation within amyloid precursor protein modulates global gene expression towards the pathogenesis of Alzheimer’s disease. *BMB Rep.* 43 704–709. 10.5483/bmbrep.2010.43.10.704 21034535

[B94] Singh S. Yu S. Yu U. Jo S. Ahn J. (2019). Low-cost solution for rodent home-cage behaviour monitoring. *PLoS One* 14:0220751. 10.1371/journal.pone.0220751 31374097 PMC6677321

[B95] Skinner B. F. (1937). Two types of conditioned reflex: A reply to konorski and miller. *J. Gen. Psychol.* 16 272–279. 10.1080/00221309.1937.9917951

[B96] Skinner B. F. (1986). Some thoughts about the future. *J. Exp. Anal. Behav.* 45 229–235. 3958668 10.1901/jeab.1986.45-229 PMC1348231

[B97] Staddon J. E. R. Cerutti D. T. (2003). Operant conditioning. *Annu. Rev. Psychol.* 54 115–144. 10.1146/annurev.psych.54.101601.145124 12415075 PMC1473025

[B98] Sullivan J. A. (2022). Novel tool development and the dynamics of control: The rodent touchscreen operant chamber as a case study. *Philos. Sci.* 89 1203–1212. 10.1017/psa.2022.63

[B99] Sutoko S. Masuda A. Kandori A. Sasaguri H. Saito T. Saido T. (2021). Early identification of Alzheimer’s disease in mouse models: Application of deep neural network algorithm to cognitive behavioral parameters. *iScience* 24 102198. 10.1016/j.isci.2021.102198 33733064 PMC7937558

[B100] Talpos J. C. Winters B. D. Dias R. Saksida L. M. Bussey T. J. (2009). A novel touchscreen-automated paired-associate learning (PAL) task sensitive to pharmacological manipulation of the hippocampus: A translational rodent model of cognitive impairments in neurodegenerative disease. *Psychopharmacology* 205 157–168. 10.1007/s00213-009-1526-3 19357840

[B101] Uddin L. Q. (2021). Cognitive and behavioural flexibility: neural mechanisms and clinical considerations. *Nat. Rev. Neurosci*. 22, 167–179. 10.1038/s41583-021-00428-w 33536614 PMC7856857

[B102] Upîte J. Kadish I. van Groen T. Jansone B. (2020). Subchronic administration of auranofin reduced amyloid-β plaque pathology in a transgenic APPNL-G-F/NL-G-F mouse model. *Brain Res*. 1746. 10.1016/j.brainres.2020.147022 32707043

[B103] Valerius G. Lumpp A. Kuelz A. Freyer T. Voderholzer U. (2008). Reversal learning as a neuropsychological indicator for the neuropathology of obsessive compulsive disorder? A behavioral study. *J. Neuropsychiatry Clin. Neurosci*. 20 210–218. 10.1176/jnp.2008.20.2.210 18451192

[B104] Van den Broeck L. Hansquine P. Callaerts-Vegh Z. D’Hooge R. (2019). Impaired reversal learning in APPPS1-21 mice in the touchscreen visual discrimination task. *Front. Behav. Neurosci.* 13:92. 10.3389/fnbeh.2019.00092 31143103 PMC6521801

[B105] Walker L. C. Lynn D. G. Chernoff Y. O. (2018). A standard model of Alzheimer’s disease? *Prion* 12 261–265. 10.1080/19336896.2018.1525256 30220236 PMC6277193

[B106] Wang H. Sun N. Wang X. Han J. Zhang Y. Huang Y. (2022). A touchscreen-based paradigm to measure visual pattern separation and pattern completion in mice. *Front. Neurosci.* 16:947742. 10.3389/fnins.2022.947742 36090275 PMC9449699

[B107] Wang W. Schuette P. Nagai J. Tobias B. Cuccovia V. Reis F. (2021). Coordination of escape and spatial navigation circuits orchestrates versatile flight from threats. *Neuron* 109 1848–1860.e8. 10.1016/j.neuron.2021.03.033. 33861942 PMC8178241

[B108] Weiss S. J. (1972). Stimulus compounding in free-operant and classical conditioning. A review and analysis. *Psychol. Bull.* 78 189–208. 10.1037/h0032956 4560788

[B109] Wetzel M. C. (1986). Operant conditioning in motor and neural integration. *Neurosci. Biobehav. Rev.* 10 387–429. 10.1016/0149-7634(86)90004-7 3543755

[B110] Wiesbrock C. Musall S. Kampa B. M. (2022). A flexible Python-based touchscreen chamber for operant conditioning reveals improved visual perception of cardinal orientations in mice. *Front. Cell. Neurosci.* 16:866109. 10.3389/fncel.2022.866109 36299493 PMC9588922

[B111] Winters B. D. Saksida L. M. Bussey T. J. (2008). Object recognition memory: Neurobiological mechanisms of encoding, consolidation and retrieval. *Neurosci. Biobehav. Rev.* 32 1055–1070. 10.1016/j.neubiorev.2008.04.004 18499253

[B112] Wobrock T. Ecker U. Scherk H. Schneider-Axmann T. Falkai P. Gruber O. (2009). Cognitive impairment of executive function as a core symptom of schizophrenia. *World J. Biol. Psychiatry* 10 442–451. 10.1080/15622970701849986 18609418

[B113] Wright A. A. Cook R. Rivera J. Sands S. Delius D. (1988). Concept learning by pigeons: Matching-to-sample with trial-unique video picture stimuli. *Anim. Learn. Behav.* 16 436–444. 10.3758/BF03209384

